# Cost Variation and Affordability of Oral Targeted Cancer Therapy in India: Comparison of Branded and Janaushadhi Products

**DOI:** 10.7759/cureus.100287

**Published:** 2025-12-28

**Authors:** Samiksha M Lohi, Chaitali A Chindhalore, Ganesh Dakhale, Snehlata Gajbhiye

**Affiliations:** 1 Pharmacology, All India Institute of Medical Sciences, Nagpur, Nagpur, IND

**Keywords:** anti-cancer medications, chronic myeloid leukemia, cost variation analysis, non-small cell lung cancer, pharmacoeconomics

## Abstract

Background

The rising cancer burden in India has increased the need for effective and affordable treatment. Over the past two decades, oral targeted therapies such as TKIs (tyrosine kinase inhibitors), PARP (poly(ADP-ribose) polymerase) inhibitors, CDK4/6 (cyclin-dependent kinase 4/6) inhibitors, and BCR-ABL (breakpoint cluster region-Abelson) inhibitors have transformed cancer care but remain highly expensive, leading to significant out-of-pocket costs. Although the Pradhan Mantri Bhartiya Janaushadhi Pariyojana (PMBJP) provides low-cost generic medicines, coverage of targeted therapies remains limited. Evaluating cost variation and affordability across branded and Janaushadhi formulations is, therefore, essential for guiding rational prescribing and policy decisions.

Methodology

This cross-sectional descriptive study was conducted over a period of six months using publicly available price data. Drug prices were extracted primarily from the Current Index of Medical Specialties (CIMS 2024-2025), which listed 33 oral targeted anticancer drugs meeting the inclusion criteria. Janaushadhi prices were obtained from the PMBJP portal. For each drug, the maximum and minimum brand prices, the median equivalent 28-day costs, cost ratios, and the percentage cost variation were calculated. Affordability was computed as the percentage of national per capita monthly income required to purchase a 28-day treatment course. A statistical comparison between branded and Janaushadhi affordability was performed using the Wilcoxon signed-rank test.

Results

A total of 33 oral targeted anticancer drugs were analyzed, of which TKIs constituted the majority (72.73%). Percentage cost variation ranged from 8% (Bosutinib) to 14,774.74% (Midostaurin), with corresponding cost ratios up to 148.75. Janaushadhi formulations were available for 14 of the 33 drugs. Absolute savings versus median branded costs ranged from ₹2,968 (Dasatinib) to ₹25,270 (Lapatinib), while percentage savings ranged from 58.89% (Dasatinib) to 91.32% (Imatinib). Affordability analysis revealed that branded formulations required 29.46%-3721.66% of the monthly per capita income, whereas Janaushadhi versions required only 3.44%-73.64%. Statistical analysis demonstrated a significant improvement in affordability with Janaushadhi products (W = 55.0, p = 0.002).

Conclusion

This study shows wide inter-brand price differences among oral targeted anticancer drugs in India, making many branded options poorly affordable. Janaushadhi formulations, where available, substantially improve affordability and reduce financial burden. However, limited PMBJP coverage restricts their overall benefit. Expanding Janaushadhi availability, improving price regulation, and encouraging rational prescribing are crucial to ensure equitable access to targeted cancer therapies in India.

## Introduction

Cancer continues to be a major public health challenge, ranking among the leading causes of morbidity and mortality worldwide [1]. In India, the cancer burden has been rising steadily, with lifestyle changes, aging populations, indoor/outdoor pollution, and improved diagnostic capabilities contributing to an increasing incidence of malignancies [2,3]. This growing disease load has placed immense strain on patients, families, and healthcare systems. Alongside this epidemiological shift, oncology has witnessed a major therapeutic transformation. The past two decades have seen the introduction and rapid expansion of oral targeted anticancer therapies, which act on specific molecular pathways critical to tumor growth and survival [4].

Oral targeted therapies include a diverse range of agents such as tyrosine kinase inhibitors (TKIs), poly(ADP-ribose) polymerase (PARP) inhibitors, cyclin-dependent kinase (CDK4/6) inhibitors, BCR-ABL (breakpoint cluster region-Abelson) inhibitors, and androgen receptor pathway inhibitors [5]. These drugs have redefined the management of cancers such as chronic myeloid leukemia (CML), breast cancer, lung cancer, renal cell carcinoma, and prostate cancer. Compared with conventional intravenous cytotoxic chemotherapy, they offer several advantages: improved efficacy in selected subgroups, a more favorable toxicity profile, and the convenience of oral administration. By enabling home-based therapy, these medicines reduce the need for frequent hospital visits and infusion-related costs, thereby enhancing patient autonomy and quality of life. Despite these clinical advances, affordability remains a major concern. Oral targeted therapies are among the most expensive categories of medicines currently used in oncology. Many are marketed at premium prices, and even when generic versions are available, the prices of different brands vary substantially. In India, where a large proportion of patients lack comprehensive health insurance coverage, the cost of medicines is often paid directly by families. In India, drug price regulation is governed by the Drugs (Prices Control) Order, 2013 (DPCO), under which the National Pharmaceutical Pricing Authority (NPPA) fixes ceiling prices for selected essential medicines, including some anticancer formulations; however, many newer oral targeted therapies remain outside these price-control mechanisms [6]. As a result, wide price variation among brands can strongly influence whether patients are able to initiate and continue therapy. Patients may be forced to discontinue treatment, skip doses, or switch to suboptimal alternatives due to unaffordability. Thus, price variation is not merely an economic issue but also a determinant of treatment outcomes and equity in cancer care.

To address the issue of affordability, the Government of India launched the Pradhan Mantri Bhartiya Janaushadhi Pariyojana (PMBJP), popularly known as the Janaushadhi scheme. This initiative aims to provide quality generic medicines at affordable prices through Janaushadhi Kendras across the country. While the scheme has demonstrated substantial cost savings for several essential drugs, the representation of newer anticancer agents, particularly oral targeted therapies, remains limited. Evaluating the extent to which Janaushadhi can improve affordability in oncology is therefore of great importance.

Cost-variation analysis studies are cross-sectional comparisons of the maximum and minimum prices of different brands of the same drug, expressed as a cost ratio or percentage variation [7,8]. Such analyses highlight inequities in drug pricing and provide evidence to support rational prescribing and policy interventions. A few Indian studies, like Kolasani et al. [7], Adwal et al. [8], Patil et al. [9], Krishna et al. [10], and Patil et al. [11], have explored cost variation among anticancer drugs, but most have concentrated on older cytotoxic agents or broad groups of essential medicines, with only limited representation of oral targeted therapies. While these analyses demonstrated striking differences in brand prices, they do not adequately reflect the current therapeutic landscape, in which targeted therapies are increasingly first-line or maintenance treatments.

In oncology, where lifelong or prolonged therapy is often required, even small daily price differences can add up to a heavy financial burden for patients and families over time. By exposing these differences, cost-analysis studies demonstrate how patients may be overpaying based on brand selection rather than therapeutic superiority. Focused analyses dedicated to newer oral targeted agents remain scarce, underscoring the need for updated evaluations in this clinically important and rapidly expanding area of cancer treatment in India.

Beyond clinical practice, such data also serve as a baseline for evaluating interventions such as price regulation, generic substitution, or the inclusion of drugs in government schemes such as Janaushadhi. Policymakers can identify drugs where regulatory action is warranted, clinicians can counsel patients more effectively, and patients may be empowered to choose affordable options. Given this background, the present study was designed to systematically evaluate the cost and affordability of oral targeted anticancer drugs available in India. The primary objective was to conduct a cost-variation analysis of oral targeted anticancer agents by comparing inter-brand price differences and estimating standardized 28-day (monthly) treatment costs. The secondary objectives were to compare the prices of drugs listed in the Current Index of Medical Specialties (CIMS) with those available under the PMBJP scheme to determine potential cost savings, to assess the affordability of selected agents in relation to the national per capita monthly income, and to statistically evaluate differences in affordability between branded and Janaushadhi formulations.

## Materials and methods

This was a cross-sectional, descriptive cost-variation analysis conducted over six months (May 2025 to October 2025). The study utilized secondary data from publicly available sources and did not involve human participants; therefore, exemption from ethics review was obtained from the Institutional Ethics Committee (IEC Reference number: IEC/Pharmac/2025/1669).

Data on oral targeted anticancer drugs were obtained from the online CIMS (2024-2025 edition) [12], which served as the primary source for brand names and prices, as it provides manufacturer-declared maximum retail prices (MRPs). Prices were extracted during the period July-September 2025. All oral targeted anticancer drugs listed in CIMS that met the inclusion criteria were included, yielding a total of 33 drugs. When price information for a particular brand was not available in CIMS, the data were cross-verified using authentic online pharmacy portals, first from Tata 1 mg [13] and, if unavailable, from Apollo Pharmacy [14], and the listed MRP for the same brand, strength, and pack size was recorded. Whenever CIMS listed the MRP, that value was treated as the reference; online pharmacy prices were used only when the CIMS price was missing.

Information regarding the availability and pricing of government-subsidized generic formulations was extracted from the PMBJP portal [15]. Standard adult dosing for each drug was identified based on product monographs and guideline recommendations from the National Comprehensive Cancer Network (NCCN)/European Medicines Agency (EMA)/United States Food and Drug Administration (US FDA)/manufacturer label. As the aim was a molecule-level cost analysis, standard monotherapy dosing for the most common indication was used; combination regimens were not included.

Drugs were included if at least one marketed brand with an available MRP was listed in CIMS, or if missing, the brand's price could be retrieved from authenticated supplementary sources such as Tata 1mg or Apollo Pharmacy. Injectable anticancer drugs, biologics, biosimilars, and experimental therapies were excluded, along with drugs not primarily indicated for oncology or those for which no price information could be obtained from either CIMS or supplementary sources.

For each drug, data were recorded on generic name, common indication, recommended daily dose and frequency, number of brands, manufacturers, and maximum and minimum retail prices (MRPs). Drugs were included if price information was available in CIMS or verified from supplementary sources. Injectable anticancer agents, biologics, biosimilars, experimental therapies, and drugs without available price data were excluded.

Prices were standardized using a uniform cost conversion method. The price per tablet was calculated from the pack MRP and pack size, followed by the computation of cost per day and cost per 28 days. For cyclical regimens (e.g., Palbociclib 21 days on/7 days off), the actual number of tablets used in a 28-day period was considered. For drugs with variable cycles (e.g., 21- or 42-day regimens), costs were standardized to a 28-day equivalent.

The following outcome measures were derived: Cost ratio = Maximum ÷ Minimum price, Percentage cost variation = ((Maximum - Minimum) ÷ Minimum) × 100. For assessing affordability, the affordability percentage was computed using the formula: (Median equivalent cost per 28 days ÷ National per-capita monthly income) × 100. This approach expresses the proportion of monthly income required to purchase a 28-day treatment course and has been adapted from a previous study by Faruqui et al. [16]. The most recent national per capita monthly income (FY 2024-2025) was obtained from the Ministry of Statistics and Programme Implementation (MoSPI) [17].

In addition, for drugs available under the PMBJP scheme, absolute savings and percentage savings compared to branded formulations were calculated to estimate the potential cost advantage of generic substitution using the formulae: Absolute savings vs. Median/Max branded cost (₹) = Median/Max branded cost - Janaushadhi cost; Percentage savings vs. Median/Max branded cost (%) = ((Median/Max branded cost - Janaushadhi cost) ÷ Median branded cost) × 100.

Statistical analysis

The Wilcoxon signed-rank test was used to compare affordability between branded and Janaushadhi formulations, as the data were paired and non-normally distributed. All data were entered and analyzed in Microsoft Excel (2019) (Microsoft Corporation, Redmond, Washington). Descriptive statistics were presented as minimum, maximum, median, and interquartile range (IQR), and results were expressed as equivalent cost per 28 days for comparability across drugs. Statistical analysis was performed using GraphPad InStat version 3.06 (GraphPad Software, San Diego, California).

## Results

A total of 33 oral targeted anticancer drugs were analyzed, belonging to classes such as TKIs, CDK4/6 inhibitors, PARP inhibitors, and others. Table 1 shows the class-wise distribution of oral targeted anticancer drugs, with their common indications and daily doses and frequencies. TKIs represented the largest drug class, accounting for nearly two-thirds (n=24, 72.73%) of the total drugs analyzed, followed by CDK4/6 inhibitors (n=3, 9.09%). Among the TKIs, the multitargeted TKIs were the most frequent (n=8, 24.24%), followed by BCR-ABL TKI and EGFR TKI (n=3, 9.09% each). After TKIs, CDK4/6 inhibitors (n=3, 9.09%) were most common. The most frequent indications were non-small cell lung cancer (NSCLC) (n=7, 21.21%), followed by breast cancer (n=5, 15.15%) and CML (n=4, 12.12%).

**Table 1:** Class-wise distribution of oral targeted anticancer drugs with their common indication and daily dose and frequency ALK – Anaplastic Lymphoma Kinase; AML – Acute Myeloid Leukemia; BCL-2 – B-Cell Lymphoma 2; BCR-ABL TKI – Breakpoint Cluster Region–Abelson Tyrosine Kinase Inhibitor; BRAF – B-Raf Proto-Oncogene; BTK – Bruton Tyrosine Kinase; CDK4/6 – Cyclin-Dependent Kinase 4 and 6; CLL – Chronic Lymphocytic Leukemia; CLL/SLL – Chronic Lymphocytic Leukemia/Small Lymphocytic Lymphoma; CML – Chronic Myeloid Leukemia; EGFR/HER2 TKI – Epidermal Growth Factor Receptor/Human Epidermal Growth Factor Receptor-2 Tyrosine Kinase Inhibitor; ErbB – Erythroblastic Oncogene B; FLT3 – FMS-Like Tyrosine Kinase 3; GIST – Gastrointestinal Stromal Tumor; HCC – Hepatocellular Carcinoma; JAK – Janus Kinase; MCL – Mantle Cell Lymphoma; MEK – Mitogen-Activated Protein Kinase Kinase; MET – Mesenchymal-Epithelial Transition Factor (gene)/Hepatocyte Growth Factor Receptor (protein); NSCLC – Non-Small Cell Lung Cancer; PARP – Poly (ADP-Ribose) Polymerase; PI3K – Phosphatidylinositol 3-Kinase; RCC – Renal Cell Carcinoma; ROS1 – c-ros Oncogene 1; TKI – Tyrosine Kinase Inhibitor; VEGFR – Vascular Endothelial Growth Factor Receptor. Data source: Drug information (class, indication, and standard dosing) extracted from the Current Index of Medical Specialties (CIMS 2024-2025 edition) [12].

Sr. No.	Drug Class	No. of Drugs	% of Drugs	Drug Name	Common Indication	Daily Dose & Frequency
1	ALK/ROS1/MET TKI	1	3.03	Crizotinib	NSCLC	250 mg, BD
2	ALK/ROS1 TKI	1	3.03	Lorlatinib	NSCLC	100 mg, OD
3	ALK inhibitor	1	3.03	Ceritinib	NSCLC	750 mg, OD
4	BCL-2 inhibitor	1	3.03	Venetoclax	CLL	400 mg, OD
5	BCR-ABL/SRC family TKI	1	3.03	Dasatinib	CLL	100 mg, OD
6	BCR-ABL TKI	3	9.09	Bosutinib	CML	400 mg, OD
				Imatinib	CML	400 mg, OD
				Nilotinib	CML	300 mg, BD
7	BRAF inhibitor	2	6.06	Dabrafenib	Met. Melanoma	150 mg, BD
				Vemurafenib	Met. Melanoma	960 mg, BD
8	BTK inhibitor	2	6.06	Acalabrutinib	CLL, MCL	100 mg, OD
				Ibrutinib	CLL/SLL	420 mg, OD
9	CDK4/6 inhibitor	3	9.09	Abemaciclib	Breast cancer	150 mg, BD
				Palbociclib	Breast cancer	125 mg, OD
				Ribociclib	Breast cancer	600 mg, OD
10	Dual EGFR/HER2 TKI	1	3.03	Lapatinib	Breast cancer	1250 mg, OD
11	EGFR/ErbB family TKI (irreversible)	1	3.03	Afatinib	NSCLC	40 mg, OD
12	EGFR TKI	3	9.09	Erlotinib	NSCLC	150 mg, OD
				Gefitinib	NSCLC	250 mg, OD
				Osimertinib	NSCLC	80 mg, OD
13	JAK inhibitor	1	3.03	Ruxolitinib	Myelofibrosis	15 mg, BD
14	MEK inhibitor	1	3.03	Trametinib	Met. Melanoma	2 mg, OD
15	Multitargeted TKI	8	24.24	Cabozantinib	Met. RCC	60 mg, OD
				Lenvatinib	Thyroid cancer	24 mg, OD
				Pazopanib	Adv. RCC	800 mg, OD
				Regorafenib	GIST	160 mg, OD
				Sorafenib	HCC	400 mg, BD
				Sunitinib	Met. RCC	50 mg, OD
				Midostaurin	FLT3-mutated AML	50 mg, BD
				Nintedanib	Idiopathic pulmonary fibrosis	150 mg, BD
16	PARP-1 and PARP-2 inhibitor	1	3.03	Olaparib	Epithelial Ovarian, Fallopian Tube, or Primary Peritoneal Cancer	300 mg, BD
17	PI3K inhibitor	1	3.03	Alpelisib	Breast cancer	300 mg, OD
18	VEGFR TKI	1	3.03	Axitinib	RCC	5 mg, BD
	TOTAL	33	100	-	-	-

Table 2shows cost variation among different brands of oral targeted anticancer drugs. Marked variation in the cost of different brands of oral targeted anticancer drugs was observed. The number of brands per molecule ranged from 1 to 33. Drugs such as Gefitinib (33 brands) and Imatinib (31 brands) had the highest market availability, whereas several drugs (e.g., Abemaciclib, Ribociclib) were available from only a single manufacturer. The cost variation percentage ranged widely from 8% (Bosutinib) to 14774.74% (Midostaurin), with a corresponding cost ratio ranging from 1.08 to 148.75. The highest cost variation was observed for Midostaurin (14,774.74%), followed by Sorafenib and Osimertinib.

**Table 2:** Cost variation among different brands of oral targeted anticancer drugs NA – Not applicable, as only a single manufacturing brand was there. Data Source: Price data obtained from CIMS (2024-2025 edition) and Tata 1 mg/Apollo Pharmacy [12-14].

Sr. No.	Drug Name	No. of Brands	Maximum Equivalent Cost/28 Days (₹)	Minimum Equivalent Cost/28 Days (₹)	Median Equivalent Cost/28 Days (₹)	Cost Variation (%)	Cost Ratio
1.	Abemaciclib	1	95480	95480	95480	NA	NA
2.	Acalabrutinib	3	28952	24472	28952	18.31	1.18
3.	Afatinib	4	58996	5314	5586	1010.2	11.1
4.	Alpelisib	1	72016	72016	72016	NA	NA
5.	Axitinib	5	166936	5992	9240	2685.98	27.86
6.	Bosutinib	2	8316	7700	8008	8	1.08
7.	Cabozantinib	6	21546	11970	13048	80	1.8
8.	Ceritinib	2	70000	20748	70000	237.38	3.37
9.	Crizotinib	1	636776	636776	636776	NA	NA
10.	Dabrafenib	1	169848	169848	169848	NA	NA
11.	Dasatinib	9	184072	2912	5040	6221.15	63.21
12.	Erlotinib	21	112840	2492	9240	4428.09	45.28
13.	Gefitinib	33	26600	1344	9520	1879.17	19.79
14.	Ibrutinib	2	306096	26628	166362	1049.53	11.5
15.	Imatinib	31	14000	1400	6776	900	10
16.	Lapatinib	9	62300	27720	37870	124.75	2.25
17.	Lenvatinib	5	180096	12880	29792	1298.26	13.98
18.	Lorlatinib	2	273000	20748	146874	1215.79	13.16
19.	Midostaurin	5	5985000	40236	80808	14774.74	148.75
20.	Nilotinib	2	117600	87528	99848	34.36	1.34
21.	Nintedanib	7	145544	5040	5684	2787.78	28.88
22.	Olaparib	13	612640	21728	28448	2719.59	28.2
23.	Osimertinib	2	458136	7644	232890	5893.41	59.93
24.	Palpociclib	5	95004	7613	30009	1147.92	12.48
25.	Pazopanib	3	32424	7448	18648	335.34	4.35
26.	Regorafenib	3	177912	29736	126588	498.31	5.98
27.	Ribociclib	1	67473	67473	67473	NA	NA
28.	Ruxolitinib	1	226184	226184	226184	NA	NA
29.	Sorafenib	10	272608	3920	8512	6854.29	69.54
30.	Sunitinib	7	162680	4667	5189	3385.75	34.86
31.	Trametinib	1	181076	181076	181076	NA	NA
32.	Vemurafenib	1	56000	56000	56000	NA	NA
33.	Venetoclax	1	383488	383488	383488	NA	NA

Table 3 presents drugs with Janaushadhi availability and cost-savings analysis. Out of the 33 studied drugs, Janaushadhi formulations were available for 14 drugs (42.4%). The 28-day cost of these formulations was considerably lower than that of their branded equivalents. Absolute savings relative to the median branded cost ranged from ₹ 2,968 (Dasatinib) to ₹25,270 (Lapatinib), while percentage savings ranged from 58.89% (Dasatinib) to 91.32% (Imatinib). The maximum percentage savings relative to the highest-priced brand equivalents were observed with Sunitinib (99.24%), followed by Sorafenib (98.97%) and Dasatinib (98.87%).

**Table 3:** Janaushadhi availability of drugs and cost savings analysis *NA – Not available. The drug is listed under Janaushadhi, but the price had not been published/finalized (recorded as ₹0) at the time of data extraction; therefore, savings could not be calculated. Data Source: Price data obtained from CIMS (2024-2025 edition) and the Janaushadhi (PMBJP) portal [12,15].

Sr. No.	Drug Name	Janaushadhi Equivalent Cost/28 Days (₹)	Branded Median Equivalent Cost/28 Days (₹)	Absolute Saving vs. Median Branded Cost (₹)	Absolute Saving vs. Max Branded Cost (₹)	% Saving vs. Median Branded Cost	% Saving vs. Max Branded Cost
1.	Cabozantinib	3332	13048	9716	18214	74.46	84.54
2.	Crizotinib*	NA	636776	NA	NA	NA	NA
3.	Dasatinib	2072	5040	2968	182000	58.89	98.87
4.	Erlotinib	1848	9240	7392	110992	80	98.36
5.	Gefitinib	1148	9520	8372	25452	87.94	95.68
6.	Ibrutinib*	NA	166362	NA	NA	NA	NA
7.	Imatinib	588	6776	6188	13412	91.32	95.8
8.	Lapatinib	12600	37870	25270	49700	66.73	79.78
9.	Lenvatinib*	NA	29792	NA	NA	NA	NA
10.	Nintedanib	2240	5684	3444	143304	60.59	98.46
11.	Olaparib*	NA	28448	NA	NA	NA	NA
12.	Pazopanib	5432	18648	13216	26992	70.87	83.25
13.	Sorafenib	2800	8512	5712	269808	67.11	98.97
14.	Sunitinib	1232	5189	3957	161448	76.26	99.24

Table 4 shows the affordability of oral targeted anticancer drugs. Affordability was calculated as the percentage of monthly per capita income required to purchase a 28-day treatment course. Among branded formulations, affordability values ranged from 29.46% (Dasatinib) to 3,721.66% (Crizotinib). For the 14 drugs available as Janaushadhi formulations, affordability percentages ranged from 3.44% (Imatinib) to 73.64% (Lapatinib).

**Table 4:** Affordability of oral targeted anticancer drugs NA – Not applicable, as the drug price is not available. Data Source: Price data extracted from CIMS (2024–2025 edition) and the PMBJP (Janaushadhi) portal [12,15]. National per capita monthly income obtained from the Ministry of Statistics and Programme Implementation (FY 2024–2025) [17].

Sr. No.	Drug Name	Branded Drug Affordability (%)	Janaushadhi Drug Affordability (%)
				1.	Abemaciclib	558.04	NA
2.	Acalabrutinib	169.21	NA
3.	Afatinib	32.65	NA
4.	Alpelisib	420.9	NA
5.	Axitinib	54	NA
6.	Bosutinib	46.8	NA
7.	Cabozantinib	76.26	19.47
8.	Ceritinib	409.12	NA
9.	Crizotinib	3721.66	NA
10.	Dabrafenib	992.68	NA
11.	Dasatinib	29.46	12.11
12.	Erlotinib	54	10.8
13.	Gefitinib	55.64	6.71
14.	Ibrutinib	972.31	NA
15.	Imatinib	39.6	3.44
16.	Lapatinib	221.33	73.64
17.	Lenvatinib	174.12	NA
18.	Lorlatinib	858.41	NA
19.	Midostaurin	472.29	NA
20.	Nilotinib	583.57	NA
21.	Nintedanib	33.22	13.09
22.	Olaparib	166.27	NA
23.	Osimertinib	1361.13	NA
24.	Palpociclib	175.39	NA
25.	Pazopanib	108.99	31.75
26.	Regorafenib	739.85	NA
27.	Ribociclib	394.35	NA
28.	Ruxolitinib	1321.94	NA
29.	Sorafenib	49.75	16.36
30.	Sunitinib	30.33	7.2
31.	Trametinib	1058.31	NA
32.	Vemurafenib	327.29	NA
33.	Venetoclax	2241.31	NA

**Figure 1:**
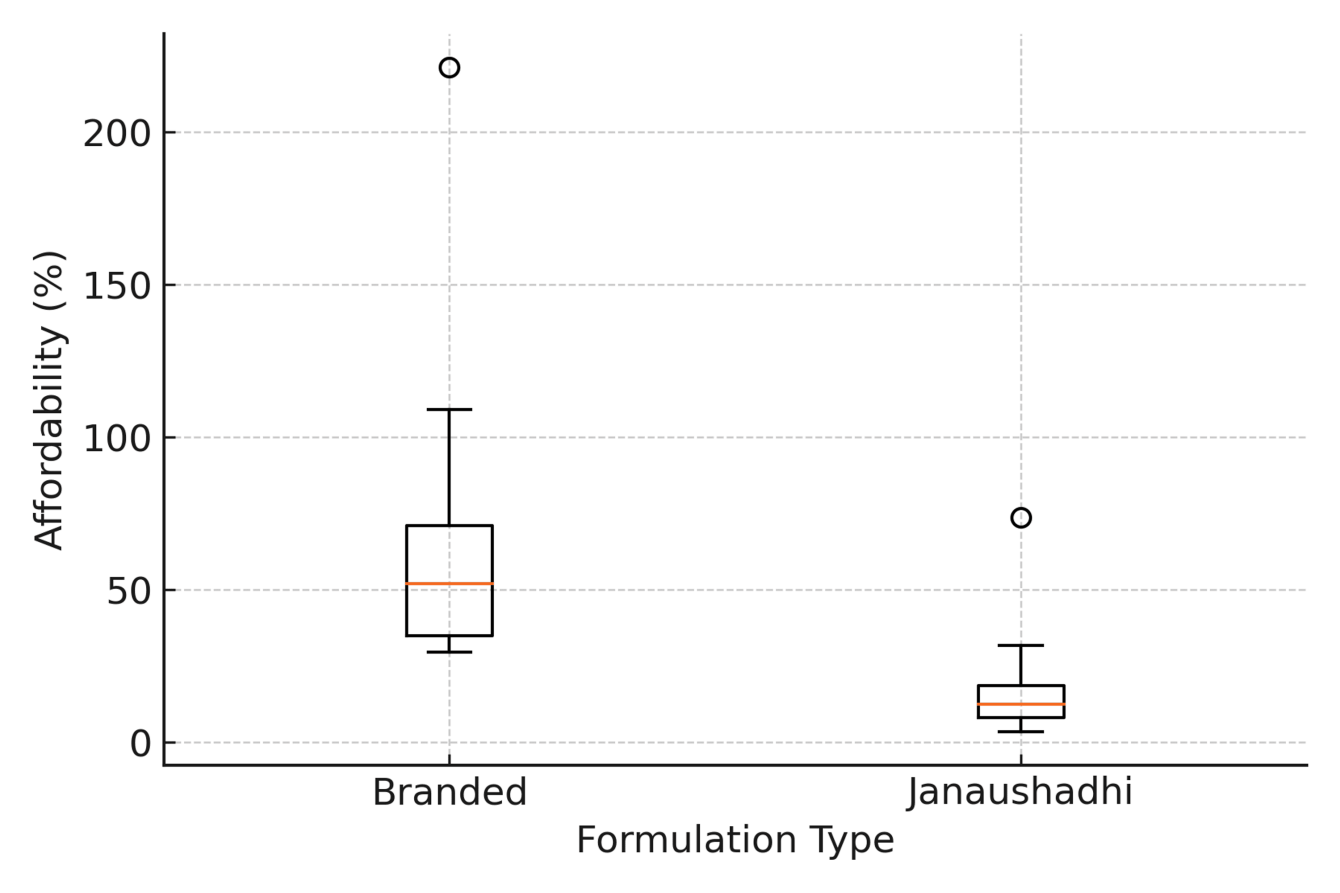
Affordability comparison between branded and Janaushadhi drugs. Note: Boxes show the interquartile range (IQR), the horizontal line inside each box indicates the median, whiskers represent variability within 1.5×IQR, and dots represent outliers. Affordability differed significantly between branded and Janaushadhi formulations (Wilcoxon W = 55, p = 0.002).

Figure 1 shows the comparison in affordability between branded and Janaushadhi formulations using the Wilcoxon matched-pairs signed-rank test. The median affordability of branded formulations was 51.88% (IQR 29.46-221.33), while that of Janaushadhi formulations was 12.60% (IQR 3.44-73.64). The difference was statistically significant (W = 55.0, p = 0.002) with a large effect size (r ≈ 0.98).

## Discussion

The rapid evolution of oncology over the past two decades has been marked by a steady shift from conventional cytotoxic chemotherapy to targeted oral therapies. These agents have become central to the management of several malignancies, offering improved outcomes and patient convenience. Globally, their use has expanded sharply. Fu et al. reported increased utilization of 44 oral targeted anticancer drugs between 2011 and 2018, while Moreira et al. observed a multi-fold rise in their use for lung and hematological cancers [18,19]. Despite this growth, access and affordability remain critical barriers in low- and middle-income countries such as India. The present study addresses this gap by systematically evaluating the cost variation, affordability, and Janaushadhi scheme coverage of oral targeted anticancer drugs marketed in India.

In the present study, 33 oral targeted anticancer drugs were analyzed across various classes, including TKIs, CDK4/6 inhibitors, PARP inhibitors, and others. TKIs represented the largest drug class in our dataset, followed by CDK4/6 inhibitors (Table 1). Among the TKIs, the multitargeted TKIs were mostly indicated for solid tumors, including thyroid cancer, renal cell carcinoma (RCC), gastrointestinal stromal tumor (GIST), and hepatocellular carcinoma (HCC), consistent with published evidence, whereas Midostaurin was indicated for hematological malignancy (acute myeloid leukemia). These patterns collectively reflect current global and national treatment trends, confirming that oral TKIs constitute the backbone of modern targeted therapy across both solid and hematological malignancies [4,20].

In our study, NSCLC and CML were the most frequent indications for oral targeted anticancer drugs. This finding aligns with global therapeutic trends. According to the study by Koban et al., small-molecule TKIs constitute the majority of targeted agents and remain the most widely used targeted oral therapies in NSCLC [21]. Similarly, for CML, four oral BCR-ABL TKIs (imatinib, dasatinib, nilotinib, and bosutinib) remain the primary agents worldwide for frontline management of CML, consistent with the observations of Senapati et al. [22]. These findings explain the high representation of NSCLC- and CML-directed agents among oral targeted anticancer drugs in the present analysis.

The number of brands per molecule ranged from 1 to 33, with Gefitinib and Imatinib having the greatest market representation. A study done by Kolasani et al. also documented wide disparities in brand availability across anticancer agents [7]. In our analysis, recently approved and patented drugs such as Abemaciclib and Ribociclib were available from a single manufacturer. This pattern aligns with global market behavior, in which brand multiplicity is primarily driven by patent expirations and subsequent generic entry [23].

Cost variation across brands was substantial, ranging from 8% (Bosutinib) to 14,774.74% (Midostaurin), with cost ratios up to 148.75. Although our analysis focused solely on oral targeted anticancer drugs, comparable inter-brand disparities have been observed in broader Indian cost-variation studies. A study done by Patil et al. reported variations ranging from 40% to over 840,619% across multiple anticancer classes, while Kolasani et al. documented differences of up to 714.24% among 23 anticancer drugs [7,11]. These results together highlight that wide cost differences persist in the Indian oncology market regardless of therapeutic class. Similar observations were noted by Krishna et al., who explained that high inter-brand price differences often arise from dependence on imported raw materials, brand monopolies, and limited government control over the pricing of oncology medicines [10].

The largest price variation in our study was observed for Midostaurin, followed by Sorafenib. Midostaurin, a relatively recently introduced multitargeted FLT3 inhibitor used in acute myeloid leukemia, showed the highest cost variation despite being marketed by five companies. This unusually wide range cannot be attributed solely to limited competition, as several other recently approved drugs with fewer manufacturers demonstrated lower variation in our study. Rather, this variation probably results from several factors, such as the limited clinical use of the drug, the absence of DPCO (Drug Prices Control Order) price control, and the presence of a high-priced innovator brand that widens the overall cost gap. These findings suggest that market maturity alone does not determine price stability; drug-specific demand, regulatory coverage, and manufacturer pricing strategy also play important roles.

Although Sorafenib is now off-patent, it still demonstrated a substantial inter-brand price gap in our analysis. This finding is consistent with observations by Kolasani et al., who also reported marked cost variation for Sorafenib in the Indian market [7]. In our study, persistent price differences even among older molecules such as Imatinib and Gefitinib suggest that prescriber preference and patient brand perception continue to sustain higher-priced products. These findings imply that both market maturity and behavioral economics influence brand pricing: older drugs develop brand loyalty, while newer ones exploit limited post-patent competition.

Out of 33 drugs, only 14 were available through the PMBJP. A study by Krishna et al. has highlighted the importance of expanding the availability of affordable oncology drugs through the Janaushadhi scheme to improve accessibility and reduce financial burden on patients [10]. The limited inclusion of oral targeted agents in the Janaushadhi scheme is likely due to challenges such as low production volumes, complex manufacturing processes, and unresolved patent issues. Expanding this formulary could substantially reduce patient expenses, given the substantial price gaps observed when Janaushadhi versions are available for branded equivalents.

Absolute savings over the median branded cost ranged from ₹2968 (Dasatinib) to ₹25,270 (Lapatinib), whereas the percentage savings over the median branded cost ranged from 58.89% (Dasatinib) to 91.32% (Imatinib). Studies by Krishna et al. and George et al. used related metrics such as cost difference and cost variation and similarly found large reductions when drugs from the Janaushadhi scheme were substituted for branded drugs [10,24]. Collectively, these findings indicate that wherever Janaushadhi alternatives are available, they provide substantial financial relief from high branded‐drug expenditure. However, several drugs in our analysis (e.g., Crizotinib, Ibrutinib, Lenvatinib, Olaparib) had no finalized Janaushadhi scheme prices, indicating gaps in PMBJP data publication.

Affordability analysis using the proportion of monthly per capita income required for a 28-day treatment showed that branded formulations demanded between 29.46% (Dasatinib) and 3721.66% (Crizotinib) of income, indicating catastrophic out-of-pocket expenditure for many targeted drugs. Wherever Janaushadhi formulations were available, affordability improved dramatically, ranging from 3.44% to 73.64%. A systematic review done by Ocran Mattila et al. reported that anticancer medicines remain among the least affordable treatments in low- and middle-income countries, highlighting the persistent economic barriers to cancer care [25]. These findings emphasize that lower Janaushadhi prices directly ease the financial burden on patients, making life-saving cancer therapy more attainable.

The affordability comparison was statistically significant (Figure 1), with a substantial effect size (r ≈ 0.98), confirming a real and consistent difference between branded and Janaushadhi formulations. The significant reduction in median affordability, from 51.88% to 12.60%, illustrates how generic availability can substantially improve affordability and expand patient access. From a public health perspective, such differences can translate into better treatment adherence and survival outcomes, consistent with previous studies [10,24] suggesting that cost minimization through PMBJP substitution can improve therapy continuation in cancer care and equity by enhancing access among lower-income populations. Our findings provide quantitative evidence supporting these observations and highlight the real-world value of improving access to affordable cancer medicines.

From a policy perspective, the wide inter-brand price variation observed in our study indicates the need for stronger pricing oversight for targeted anticancer drugs. Improving transparency in brand-wise MRP reporting, considering price-regulation mechanisms for selected high-cost targeted cancer therapies, and strengthening generic substitutions may help narrow existing price gaps. Expanding the Janaushadhi formulary would further enhance affordability. Integrating pharmacoeconomic evaluation into oncology drug approval and pricing decisions could also support more rational and patient-centered cancer medicine policies in India.

Strengths of the study

A strength of our study is its comprehensive and current focus on oral targeted therapies, an area that has received limited attention in previous Indian cost-analysis literature. The analysis of both branded and Janaushadhi formulations using standardized 28-day cost equivalents allows meaningful comparison across drugs with different dosing schedules. Incorporating affordability in relation to the national per capita income adds real-world context, making the findings more interpretable for policymakers. Lastly, statistical validation of affordability differences between branded and Janaushadhi drugs strengthens the credibility of our conclusions and provides a framework for future pharmacoeconomic research on medicine affordability in India.

Limitations of the study

Our study has certain limitations. Although each drug was evaluated for its most common indication, costs were not stratified across all possible cancer types or patient-specific factors that may influence dosing. The analysis focused on the cost of individual oral targeted agents; costs of other drugs used in combination regimens were not included, as the aim was to assess molecule-level price variation and affordability. Price data were obtained only from CIMS and the Janaushadhi database; hospital procurement rates and local pharmacy variations were not included. As a cross-sectional study, the findings reflect prices at a single time point and may not account for future market or NPPA revisions. Affordability estimates were based on national per capita monthly income and did not account for state-wise or urban-rural income variations, which may affect actual affordability in lower-income regions. Despite these limitations, the study provides valuable insights into the cost and affordability of targeted cancer medicines in India.

## Conclusions

This study provides recent evidence of wide cost variation and poor affordability among oral targeted anticancer drugs in India. Although TKIs dominate current targeted therapy, their branded versions remain largely unaffordable for most patients. Availability of Janaushadhi formulations offers substantial cost savings and improved affordability, reinforcing the role of generics in reducing financial toxicity. However, the limited inclusion of targeted agents under the PMBJP scheme restricts the overall benefit. Expanding Janaushadhi coverage and strengthening price-regulation mechanisms could help ensure equitable and sustainable access to life-saving cancer treatments. Future studies could incorporate hospital procurement prices or real-world retail selling prices, evaluate regimen-level affordability for combination therapies, assess temporal price changes, and explore regional income differences to better understand variations in affordability and guide policy planning.
